# Musculoskeletal pain latent classes and biopsychosocial characteristics among emerging adults

**DOI:** 10.1186/s12891-023-06412-y

**Published:** 2023-04-28

**Authors:** Kaitlin M. Gallagher, Erin K. Howie, Makayla Carney

**Affiliations:** Exercise Science Research Center, Department of Health, Human Performance, and Recreation, University of Arkansas, Fayetteville, AR USA

**Keywords:** Latent class analysis, Young adults, Mental health, Sleep, Physical activity

## Abstract

**Background:**

Emerging adults (aged 18–29) report high levels of musculoskeletal pain; however, it is unknown if body location and intensity patterns are related to different biopsychosocial characteristics. This study identified patterns of self-reported musculoskeletal pain among emerging adults and assessed if there were differences in their lifestyle and psychological characteristics.

**Methods:**

Data from survey responses from a large public university and a large medical university in the United States were used (n = 1,318). Self-reported pain location and intensity at five body regions were assessed, and latent class analysis identified classes separately for men and women. Mental health, physical activity, and sleep outcomes were compared between the classes.

**Results:**

Four classes were identified for men and women. Three of the classes were consistent between genders – “no pain,” (women = 28% of their sample; men = 40% of their sample) “mild multisite pain,” (women = 50%; men = 39%) and “moderate-severe multisite pain” (women = 9%; men = 7%). The fourth class for women was “moderate spine pain,” (13%) and for men was “mild extremity pain” (13%). For both men and women, the “moderate-severe multisite” pain classes reported the highest levels of depression, anxiety, and stress, poorer sleep, and higher work physical activity than the “no pain” class. The “mild multisite” and “moderate spine” (women only) pain classes fell between the “no pain” and “moderate-severe” pain classes. The characteristics of the “mild upper extremity pain” class for men was similar to the “no pain” class.

**Conclusions:**

The identified classes provide unique information on pain location and intensity in emerging adults. The high prevalence of “mild multisite pain” (n = 593; 45% of the total sample) demonstrates an intervention opportunity during this age range to prevent further increases in musculoskeletal pain later in life. Future work should assess the longitudinal outcomes of these pain classes, the impact of interventions for this age group, and the balance between leisure and occupational physical activity when addressing musculoskeletal health.

**Supplementary Information:**

The online version contains supplementary material available at 10.1186/s12891-023-06412-y.

## Introduction

Musculoskeletal and mental health conditions contribute to a high proportion of years lived with disability. Globally, over one billion people live with musculoskeletal conditions (such as low back pain, neck pain, and osteoarthritis) [1], and over 900 million live with mental disorders [2]; however, many people simultaneously deal with physical and mental illnesses [3]. This relationship is documented in children and adolescents [4–8]; however, longitudinal work assessing the age group of 18–29, recognized as emerging adulthood [9–11], shows that musculoskeletal complaints increase in prevalence from adolescence into their second decade [7, 12], and physical symptoms are related to depressive states from adolescence to emerging adulthood [13]. 30% of emerging adults with low back pain in their adolescence said their pain interfered with school, activities of daily living, and recreational physical activity [14]. Given that emerging adulthood is not always assessed as a distinct age group [15] despite increasing musculoskeletal pain prevalence compared to adolescence [7, 12] and its association with mental health conditions[13], this study used latent class analysis to examine the complex relationship between musculoskeletal health and mental health disorders in 18-29-year-olds.

The International Association for the Study of Pain describes “*pain”* as “an unpleasant sensory and emotional experience associated with actual or potential tissue damage or described in terms of such damage” [16]; however, it is important to consider that multiple factors may influence a person’s experience [17]. The biopsychosocial model of pain suggests a dynamic and multidimensional relationship between biological, psychological, and social factors that must be understood to characterize a patient’s health and pain experiences [3, 18]. Because pain induces various emotional responses, these factors influence how individuals perceive pain [19]. For example, baseline depression in adolescents is related to higher pain disability and poor quality of life after four months [20].

The biopsychosocial model of pain also demonstrates the importance of considering lifestyle factors when evaluating pain. Two such factors are physical activity and sleep. First, emerging adults have a high amount of sedentary time (~ 60% of waking time) and physical inactivity [21]; however, males who maintain a regular exercise plan or adopt regular exercise have a better quality of life and depression scores than those who do not maintain a regular exercise program or stopped exercising once they reached emerging adulthood [22]. Despite the many noted benefits of physical activity, its impact on musculoskeletal pain is mixed. For example, physical activity was not associated with musculoskeletal complaints in adolescents [7], and physical activity did not mediate the effect of sleep problems on musculoskeletal severity in emerging adults after a 3-year follow-up [23]. The added complexity also arises due to the different domains of physical activity, such that there may be different physiological responses for leisure and occupational physical activity [24, 25]. Second, sleep may impact a person’s pain directly; however, it is also possible that inadequate sleep may cause depression and lead to chronic pain [26]. In emerging adults, sleep is associated with chronic and musculoskeletal pain, and it also predicts chronic pain and an increase in musculoskeletal pain after three years [23].

A final factor to consider when addressing the biopsychosocial model of pain is if the reported pain occurs at a single site or is more widespread. Pain at multiple sites (two or more) is more prevalent than single-site pain and is associated with increased psychological distress [27, 28], pain intensity [27], sleep quality [28, 29], and decreased function during activities of daily living [29]. Low back pain is comorbid with other pain conditions in children and adolescents, such as neck and shoulder pain [8]. In a longitudinal study from adolescence into emerging adulthood, 73% of respondents reported musculoskeletal pain in at least three pain sites at baseline, and reporting at least four adverse lifestyle factors doubled the odds that these individuals would report persistent multisite musculoskeletal pain after 11 years [12].

Studies typically treat widespread or multisite pain as the cumulative number of sites a person reports; however, they do not emphasize patterns in reporting and if lifestyle factors (such as sleep and physical activity) or mood disorders differ depending on the pain pattern. Latent class analysis can assist in finding underlying pain patterns by grouping unobserved subpopulations using observed variables. This statistical method has been used to determine multi-site musculoskeletal symptom classes for emerging adult-aged electronic assembly workers [30], low back pain progression from adolescence into emerging adulthood [14], and lifestyle and psychosocial factors among adolescents with low back pain [4, 31]. Thus, this study aimed to assess self-reported musculoskeletal pain location and intensity in 18-29-year-olds and determine if individuals with different pain patterns also had different psychological and lifestyle characteristics. We had two aims for this paper:

1. To identify patterns of self-reported pain among emerging adults.
2. Describe the identified classes based on anthropometrics, lifestyle, and psychological factors. We hypothesized that differences would arise between the pain classes and mental health scores, occupational physical activity and sleep quality.

## Methods

A convenience sample of two cohorts from a large public university and a large university medical system in the Southern United States were recruited between October 2018 and March 2020 via word of mouth, classroom announcements, and online emails. Participants were recruited into the larger study on health, not specifically a study about pain. The current study was a secondary analysis of this sample’s emerging adults (18–29 years). For the original study, participants were invited to respond to the survey if they were a student, faculty, or staff member between 18 and 29 years old. Participation was voluntary, but individuals from the large public university were incentivized by being put into a drawing for one of five $50 Amazon gift cards. Individuals from the large medical system were incentivized by being put into drawings for t-shirts. Informed consent was obtained from all participants before the start of the survey. Each university’s Institutional Review Board approved the study.

Eligible participants completed an anonymous online survey administered in English via Qualtrics. The survey consisted of a series of questions about their musculoskeletal pain [32], mental health [33], physical activity [34], sleep [35], and demographics.

Musculoskeletal pain was self-reported using the following question [32]: “*During the past three months, to what extent have you had pain, aching, numbness, or tingling in any of these body areas*?” The body areas in question included the hand/wrist, shoulder/neck, low back, knee, and foot. Respondents were asked to rate their pain in each area on a scale of “none,” “mild,” “moderate,” “severe,” and “extreme.” While the Nordic Pain Questionnaire [36] is commonly used, allowing individuals to report both location and intensity provided additional information for the latent class analysis.

The International Physical Activity Questionnaire (IPAQ) - long form is a 31-item questionnaire allowing participants to self-report detailed habits involving occupational physical activity, leisure-time physical activity and sedentary habits [34]. Each physical activity domain was broken down into frequency and duration for three different intensities: walking, moderate, and vigorous [34]. The specific unit of energy expenditure measured for the three domains in this study was the Metabolic Minute per week. (MET·min·wk^− 1^). The MET values used for scoring each domain were 3.3 METS for walking, 4 METS for moderate PA, and 8 METS for vigorous PA [34, 37]. To find total physical activity values for the week, MET values were multiplied by minutes completed for each intensity and then multiplied by the total number of days per week completed. The totals for each domain were summed to find the total physical activity.

Mental health was assessed using the 21-item Depression, Anxiety, and Stress Scale (DASS-21), which assesses depression, anxiety, and stress symptoms [33]. The three self-reported scales consist of 7 items, each with scores ranging from 0 to 42 [38]. Participants circled a number from 0 to 3 to indicate how much each statement applied to them over the past week, with 0 being “not at all” to 3 being “very much so” [38]. Higher scores indicate poorer mental health or higher symptoms within the specified category [38].

The Pittsburgh Sleep Quality Instrument (PSQI) was used to assess the quantity and quality of sleep for participants [35]. The PSQI is a validated 19-item questionnaire assessing seven components of sleep: quality, duration, latency (the time it takes to fall asleep), habit efficiency, disturbance, sleeping medications, and daytime dysfunctions [35]. This instrument has a global scoring scale ranging from 0 to 21; individuals with a global score of 5 or greater are considered poor sleepers [35].

Data was reviewed for missing data and duplicates. When assessing participant inclusion in our analysis, data were considered missing if respondents failed to answer the question for any pain site. The latent class analysis approach categorized individuals based on pain reporting, taking into consideration both location and intensity. This analysis is a person-centered, probabilistic form of cluster analysis used to estimate group memberships. Latent class analysis [39] was performed using LatentGOLD 5.1 software (Statistical Innovations, Arlington, MA, USA), with five ordinal indicator variables of pain severity at each body location. Gender-specific models (man and woman) were examined due to known differences in pain reporting and symptoms [20, 23, 40]. The optimal number of classes was selected using the following criteria:

1. the minimum values of the goodness of fit measures Bayes Information criteria (BIC) and Akaike’s information criteria (AIC),
2. bootstrapped P-value using 500 replications for the log-likelihood difference between models,
3. the quality of the model in terms of posterior probability diagnostics, including entropy R2 value (values closer to 1 are better),
4. classification errors,
5. the percent of iterations converging on the same solution,
6. conceptual interpretation of the meaningfulness of the solutions [39, 41],
7. probability and proportion assigned,
8. average posterior probability greater than 0.7,
9. odds of correct classification greater than five [39, 41].

Participants were assigned to the class for which they had the highest posterior probability of membership. The number of pain sites (0–5), intensity (0–4), and cumulative pain intensity across all sites were calculated as descriptive statistics for each class. To examine differences between identified classes in mental health and health behaviors, linear regressions weighted for probability of membership were used. Negative binomial regressions were used as both DASS and IPAQ outcomes resemble a count distribution.

## Results

### Demographics

After screening for age and missing data from the original sample, 1,318 total participants [women: n = 714 (54%) and men: n = 604 (46%)] were included in the latent class analysis (see Additional File 1). The average age of the remaining participants from the large public university data was 19.7 (standard deviation = 1.96) years. Respondents from the large medical system reported age groups, so included participants’ ages fell between 18 and 29 years old. The overall sample was 82% white. A summary of reported outcomes is in Table 1. The average number of reported pain sites was 1.6 (1.5), with women reporting more pain sites than men. Women reported a higher cumulative pain intensity than men (2.9 vs. 2.0, *p* < .001). Women reported greater pain intensity at the shoulder/neck, low back, and knee than men. For mental health, women reported higher symptoms of anxiety and stress. For health behaviors, only work physical activity differed between sexes, with women reporting higher amounts of work-related physical activity.

**Table 1:** Sample characteristics summary

	Total (n = 1,318)	Women (n = 714)	Men (n = 604)	p-value^d^
Cumulative Pain Intensity^a^	2.5 (2.8)	2.9 (2.9)	2.0 (2.5)	< 0.001
Number of pain sites^b^	1.6 (1.5)	1.8 (1.5)	1.4 (1.5)	< 0.001
* 0*	435 (33%)	193 (27%)	242 (40%)	< 0.001
* 1*	240 (18%)	126 (18%)	114 (19%)	
* 2*	296 (22%)	172 (24%)	124 (21%)	
* 3*	186 (14%)	124 (17%)	62 (10%)	
* 4*	89 (7%)	55 (8%)	34 (6%)	
* 5*	72 (5%)	44 (6%)	28 (5%)	
Hand pain intensity^c^	0.3 (0.7)	0.4 (0.7)	0.3 (0.7)	0.346
* None*	1,018 (77%)	545 (76%)	473 (78%)	0.224
* Mild*	177 (13%)	94 (13%)	83 (14%)	
* Moderate*	98 (7%)	63 (9%)	35 (6%)	
* Severe*	21 (2%)	11 (2%)	10 (2%)	
* Extreme*	4 (0%)	1 (0%)	3 (1%)	
Shoulder/neck pain intensity^c^	0.8 (0.9)	0.9 (1.0)	0.6 (0.8)	< 0.001
* None*	689 (52%)	320 (45%)	369 (61%)	< 0.001
* Mild*	327 (25%)	185 (26%)	142 (24%)	
* Moderate*	242 (18%)	166 (23%)	76 (13%)	
* Severe*	50 (4%)	38 (5%)	12 (2%)	
* Extreme*	10 (1%)	5 (1%)	5 (1%)	
Low back pain intensity^c^	0.7 (0.9)	0.9 (1.0)	0.5 (0.8)	< 0.001
* None*	747 (57%)	344 (48%)	403 (67%)	< 0.001
* Mild*	305 (23%)	181 (25%)	124 (21%)	
* Moderate*	199 (15)%	136 (19%)	63 (10%)	
* Severe*	51 (4%)	42 (6%)	9 (1%)	
* Extreme*	16 (1%)	11 (2%)	5 (1%)	
Knee pain intensity^c^	0.4 (0.7)	0.5 (0.8)	0.4 (0.7)	< 0.001
* None*	952 (72%)	492 (69%)	460 (76%)	0.020
* Mild*	202 (15%)	116 (16%)	86 (14%)	
* Moderate*	131 (10%)	82 (11%)	49 (8%)	
* Severe*	27 (2%)	20 (3%)	7 (1%)	
* Extreme*	6 (0%)	4 (1%)	2 (0%)	
Foot pain intensity^c^	0.3 (0.6)	0.3 (0.7)	0.3 (0.6)	0.480
* None*	1,078 (82%)	587 (82%)	491 (81%)	0.179
* Mild*	139 (11%)	65 (9%)	74 (12%)	
* Moderate*	83 (6%)	49 (6.9%)	34 (6%)	
* Severe*	14 (1%)	10 (1%)	4 (1%)	
* Extreme*	4 (0%)	3 (0%)	1 (0%)	
Depression^e^	9.2 (10.1)	9.2 (9.7)	9.2 (10.6)	0.990
Anxiety^e^	7.2 (7.9)	8.1 (8.2)	6.3 (7.5	< 0.001
Stress^e^	11.7 (9.1)	13.4 (9.1)	9.8 (8.8)	< 0.001
Total Physical Activity (METmin/wk)	3,623 (3,537)	3,477 (3,413)	3,794 (3,673)	0.112
Work Physical Activity (METmin/wk)	843 (2,082)	965 (2,109)	701 (2,043)	0.025
Leisure Physical Activity(METmin/wk)	1,383 (1,953)	1,298 (1,838)	1,482 (2,076)	0.096
Sleep^f^	5.7 (3.1)	5.8 (3.1)	5.6 (3.1)	0.313

^a^Calculated as the sum of intensity scores across pain sites (score of 0–20).

^b^The first line represents the mean (standard deviation) of the number of pain sites for each class.

^c^Rated on a score from none (0) to extreme (4). The first line represents the mean (standard deviation) of the pain intensity for each class at that region. The subsequent lines represent the distribution among the five pain intensity categories.

^d^p-value is for t-test or chi-squared comparing sexes.

^e^Scores range from 0 to 42 with higher numbers indicating higher symptoms.

^f^Sleep scores from Pittsburgh Sleep Quality Index with higher scores indicating poorer quality sleep.

### Latent class model selection

A four-class model was selected for women both men and women, with the fit statistics described in Additional File 2. Solutions with five- or six- classes had a small percentage of participants assigned to a class (1%). The four-class solutions were selected due to similar fit statistics to the three-class model, a significant boot-strapped log-likelihood *p*-value compared to the three class model, and conceptual interpretability of the four classes. Model fit statistics pertaining to women and men for models with 1–6 classes can be seen in Additional File 2.

### Pain Sites

#### Women

**Fig. 1:**
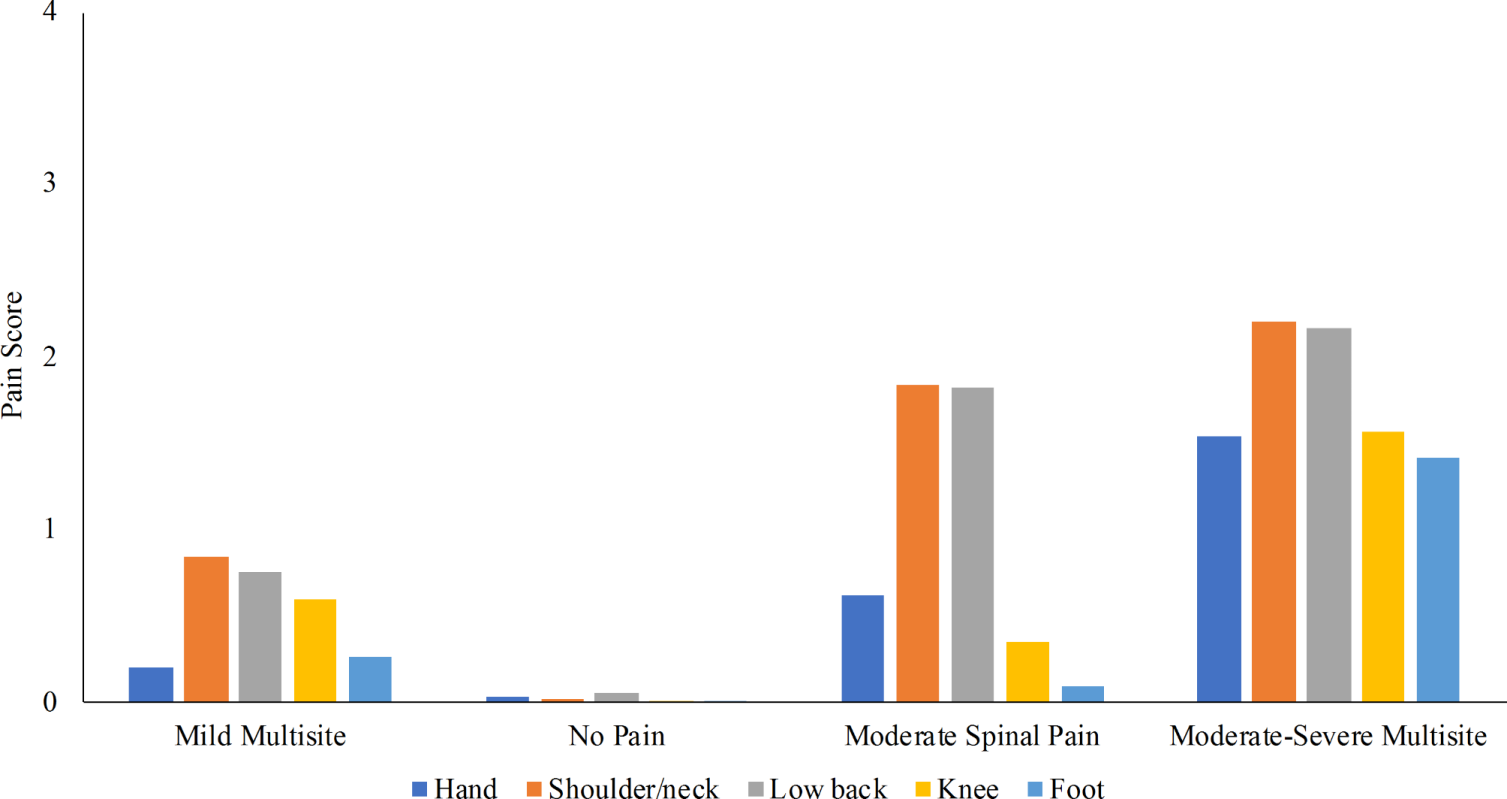
Estimated pain scores for women by latent class. The y-axis (pain score) is on a scale from 0 (no pain) to 4 (extreme pain). The x-axis is ordered left to right by the largest to smallest class size

The four classes that emerged among women were “no pain” (n = 202, 28%), “mild multisite pain” (n = 354, 50%), “moderate spine pain” (n = 95, 13%), and “moderate to severe multisite pain” (n = 63, 9%) (Fig. 1; Table 2).

For the “no pain” class, 193 members reported no symptoms (96%), and 9 reported mild hand pain (4.5%).

In the “mild multisite” class, all participants reported at least one pain site, and 67% (n = 238) reported at least two pain sites, with two pain sites being the most reported (n = 135; 38%). Mild shoulder/neck (n = 169, 48%) and low back (n = 168; 48%) pain were the most frequently reported, followed by mild knee pain (n = 83, 4%). The cumulative pain score was a mean of 2.8 (1.4).

For the “moderate spine pain” class, 94 participants (99%) reported symptoms for 2.7 (0.6) paint sites, with three pain sites being the most reported (n = 50, 53%). Moderate shoulder/neck (n = 62, 65%) and low back (n = 58, 61%) were the most reported in this class. The cumulative pain score was a mean of 5.2 (1.12).

Finally, the “moderate multisite pain” class had 100% of its members reporting at least three pain sites, and the cumulative pain score was a mean of 9.5 (2.1). Members of this class reported moderate symptoms for all pain sites compared to the “moderate spine pain” class.

Between the classes, there were no significant differences between the survey site location, average age (one site only), and BMI (one site only) (Table 2).

**Table 2:** Characteristics of each latent class for women

n = 714	Mild Multisite (n = 354; 50%)	No Pain (n = 202; 28%)	Moderate spine pain (n = 95; 13%)	Moderate-Severe Multisite (n = 63; 9%)	
Cumulative Pain Intensity^a^	2.8 (1.4)	0.04 (0.2)	5.2 (1.2)	9.5 (2.1)	
Number of pain sites^b^	2.1 (1.0)	0.04 (0.2)	2.7 (0.6)	4.5 (0.6)	
* 0*	0 (0%)	193 (95.5%)	0 (0%)	0 (0%)	
* 1*	116 (32.8%)	9 (4.5%)	1 (1.1%)	0 (0%)	
* 2*	135 (38.1%)	0 (0%)	37 (39.0%)	0 (0%)	
* 3*	69 (19.5%)	0 (0%)	50 (52.6%)	5 (7.9%)	
* 4*	27 (7.6%)	0 (0%)	7 (7.4%)	21 (33.3%)	
* 5*	7 (2.0%)	0 (0%)	0 (0%)	37 (58.7%)	
Hand pain intensity^c^	0.2 (0.5)	0.04 (0.2)	0.7 (0.9)	1.6 (0.8)	
* None*	296 (83.6%)	193 (95.5)	49 (51.6%)	7 (11.1%)	
* Mild*	42 (11.9%)	9 (4.5%)	28 (29.5%)	15 (23.8%)	
* Moderate*	14 (4.0%)	0 (0%)	14 (14.7%)	35 (55.6%)	
* Severe*	2 (0.6%)	0 (0%)	3 (3.2%)	6 (9.5%)	
* Extreme*	0 (0%)	0 (0%)	1 (1.1%)	0 (0%)	
Shoulder/neck pain intensity^c^	0.9 (0.7)	0 (0)	2.1 (0.7)	2.3 (0.7)	
* None*	116 (32.8%)	202 (100%)	2 (2.1%)	0 (0%)	
* Mild*	169 (47.7%)	0 (0%)	11 (11.6%)	5 (7.9%)	
* Moderate*	67 (18.9%)	0 (0%)	62 (65.3%)	37 (58.7%)	
* Severe*	2 (0.6%)	0 (0%)	18 (19.0%)	18 (28.6%)	
* Extreme*	0 (0%)	0 (0%)	2 (2.1%)	3 (4.8%)	
Low back pain intensity^c^	0.8 (0.7)	0 (0)	2.1 (0.9)	2.2 (0.9)	
* None*	134 (37.9%)	202 (100%)	6 (6.3%)	2 (3.2%)	
* Mild*	168 (47.5%)	0 (0%)	6 (6.3%)	7 (11.1%)	
* Moderate*	45 (12.7%)	0 (0%)	58 (61.1%)	33 (52.4%)	
* Severe*	7 (2.0%)	0 (0%)	19 (20.0%)	16 (25.4%)	
* Extreme*	0 (0%)	0 (0%)	6 (6.3%)	5 (7.9%)	
Knee pain intensity^c^	0.6 (0.9)	0 (0)	0.3 (0.5)	1.7 (1.1)	
* None*	207 (58.5%)	202 (100%)	74 (77.9%)	9 (14.3%)	
* Mild*	83 (23.5%)	0 (0%)	18 (19.0%)	15 (23.8%)	
* Moderate*	54 (15.3%)	0 (0%)	3 (3.2%)	25 (39.7%)	
* Severe*	9 (2.5%)	0 (0%)	0 (0%)	11 (17.5%)	
* Extreme*	1 (0.3%)	0 (0%)	0 (0%)	3 (4.8%)	
Foot pain intensity^c^	0.3 (0.6)	0 (0)	0.04 (0.2)	1.6 (1.1)	
* None*	281 (79.4%)	202 (100%)	91 (95.8%)	13 (20.6%)	
* Mild*	48 (13.6%)	0 (0%)	4 (4.2%)	13 (20.6%)	
* Moderate*	23 (6.5%)	0 (0%)	0 (0%)	26 (41.3%)	
* Severe*	1 (0.3%)	0 (0%)	0 (0%)	9 (14.3%)	
* Extreme*	1 (0.3%)	0 (0%)	0 (0%)	2 (3.2%)	
*n* from Public University	233 (65.8%)	146 (72.3%)	55 (57.9%)	37 (58.7%)	0.141
Age (Public only, n = 485)^d^	20.0 (2.0)	20.3 (2.3)	20.4 (1.8)	20.2 (2.1)	0.493
BMI (Public only, n = 468)^d^	24.1 (5.2)	22.9 (4.0)	23.7 (4.4)	23.9 (4.5)	0.105

^a^Calculated as the sum of intensity scores across pain sites (score of 0–20).

^b^The first line represents the mean (standard deviation) of the number of pain sites for each class.

^c^Rated on a score from none (0) to extreme (4). The first line represents the mean (standard deviation) of the pain intensity for each class at that region. The subsequent lines represent the distribution among the five pain intensity categories.

^d^Only age range was available from the medical university included in the study; therefore, age and BMI are only reported from the large public university participants.

#### Men

**Fig. 2:**
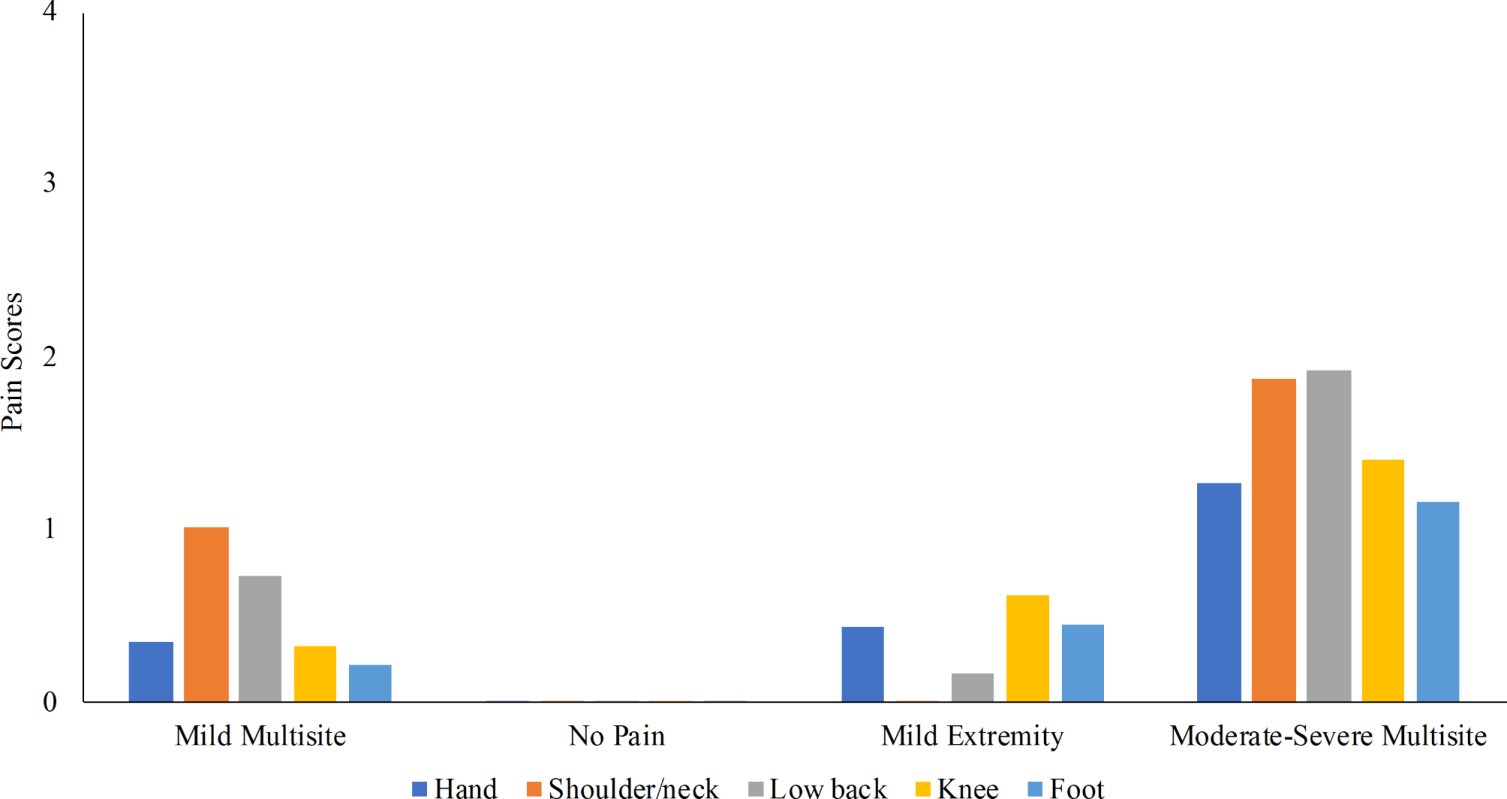
Estimated pain scores for men by latent class. The y-axis (pain score) is on a scale from 0 (no pain) to 4 (extreme pain). The x-axis is ordered left to right by the largest to smallest class size

The four classes that emerged among men were “no pain” (n = 242, 40%), “mild multisite pain” (n = 239, 40%), “Mild extremity pain” (n = 79, 13%), and “moderate-severe multisite pain” (n = 44, 7%) (Fig. 2; Table 3).

In the “mild multisite pain” class, all participants reported at least one pain site, and 75% (n = 179) reported at least two pain sites, with two pain sites being the most reported (n = 106; 44%). The cumulative pain score was a mean of 2.2 (0.9). In this class, mild shoulder/neck (n = 132, 55%) and low back (n = 110; 46%) pain were the most frequently reported, followed by mild knee pain (n = 50, 21%).

For the “mild extremity pain” class, all members reported between one and three pain sites, with one pain site being the most reported (n = 54, 68%). Mild hand (n = 19, 24%), knee (n = 43, 54%), and foot (n = 24, 30%) were the most reported sites when symptoms were reported. The cumulative pain score was a mean of 2.1 (1.1).

Finally, the “moderate-severe multisite pain” class had 98% (n = 43) of its members report report least three pain sites, and the cumulative pain score was a mean of 8.4 (2.2). Five pain sites were the most reported (n = 23, 52%). Members of this class reported moderate symptoms most frequently for the shoulder/neck (n = 25, 57%), low back (n = 25, 57%), and knee (n = 17, 39%).

Between the classes, there were no significant differences between the survey site location, average age (one site only), and BMI (one site only) (Table 3).

**Table 3:** Characteristics of each latent class for men

n = 604	Mild Multisite (n = 239; 39%)	No pain (n = 242; 40%)	Mild Extremity Pain (n = 79; 13%)	Moderate-Severe Multisite (n = 44; 7%)	
Cumulative Pain Intensity^a^	2.8 (1.4)	0 (0)	2.1 (1.1)	8.4 (2.2)	
Number of pain sites^b^	2.2 (0.9)	0 (0)	1.4 (0.7)	4.4 (0.7)	
* 0*	0 (0%)	242 (100%)	0 (0%)	0 (0%)	
* 1*	60 (25.1%)	0 (0%)	54 (68.4%)	0 (0%)	
* 2*	106 (44.4%)	0 (0%)	17 (21.5%)	1 (2.3%)	
* 3*	52 (21.8%)	0 (0%)	8 (10.1%)	2 (4.6%)	
* 4*	16 (6.7%)	0 (0%)	0 (0%)	18 (40.9%)	
* 5*	5 (2.1%)	0 (0%)	0 (0%)	23 (52.3%)	
Hand pain intensity^c^	0.4 (0.6)	0 (0)	0.6 (1.0)	1.4 (1.0)	
* None*	174 (72.8%)	242 (100%)	49 (62.0%)	8 (18.2%)	
* Mild*	47 (19.7%)	0 (0%)	19 (24.1%)	17 (38.6%)	
* Moderate*	17 (7.1%)	0 (0%)	5 (6.3%)	13 (29.6%)	
* Severe*	1 (0.4%)	0 (0%)	5 (6.3%)	4 (9.1%)	
* Extreme*	0 (0%)	0 (0%)	1 (1.3%)	2 (4.6%)	
Shoulder/neck pain intensity^c^	1.1 (0.8)	0 (0%)	0 (0%)	2.0 (0.8)	
* None*	47 (19.7%)	242 (100%)	79 (100%)	1 (2.3%)	
* Mild*	132 (55.2%)	0 (0%)	0 (0%)	10 (22.7%)	
* Moderate*	51 (21.3%)	0 (0%)	0 (0%)	25 (56.8%)	
* Severe*	6 (2.5%)	0 (0%)	0 (0%)	6 (13.6%)	
* Extreme*	3 (1.3%)	0 (0%)	0 (0%)	2 (4.6%)	
Low back pain intensity^c^	0.8 (0.8)	0 (0)	0.05 (0.2)	2.1 (0.8)	
* None*	86 (36.0)	242 (100%)	75 (94.9%)	0 (0%)	
* Mild*	110 (46.0)	0 (0%)	4 (5.1%)	10 (22.7%)	
* Moderate*	38 (15.9)	0 (0%)	0 (0%)	25 (56.8%)	
* Severe*	4 (1.7)	0 (0%)	0 (0%)	5 (11.4%)	
* Extreme*	1 (0.4)	0 (0%)	0 (0%)	4 (9.1%)	
Knee pain intensity^c^	0.3 (0.6)	0 (0)	0.8 (0.8)	1.6 (1.1)	
* None*	175 (73.2%)	242 (0%)	36 (45.6%)	7 (15.9%)	
* Mild*	50 (20.9%)	0 (0%)	24 (30.4%)	12 (27.3%)	
* Moderate*	14 (5.9%)	0 (0%)	18 (22.8%)	17 (38.6%)	
* Severe*	0 (0%)	0 (0%)	1 (1.3%)	6 (13.6%)	
* Extreme*	0 (0%)	0 (0%)	0 (0%)	2 (4.6%)	
Foot pain intensity^c^	0.2 (0.5)	0 (0)	0.6 (0.8)	1.3 (0.9)	
* None*	196 (82.0%)	242 (0%)	44 (55.7%)	9 (20.5%)	
* Mild*	35 (14.6%)	0 (0%)	24 (30.4%)	15 (34.1%)	
* Moderate*	8 (3.4%)	0 (0%)	9 (11.4%)	17 (38.6%)	
* Severe*	0 (0%)	0 (0%)	2 (2.5%)	2 (4.6%)	
* Extreme*	0 (0%)	0 (0%)	0 (0%)	1 (2.3%)	
*n* from Public University	207 (86.6%)	220 (90.9%)	72 (91.1%)	35 (79.6%)	0.214
Age (Public university only, n = 536)^d^	19.3 (1.6)	19.2 (1.5)	19.3 (1.8)	19.9 (2.8)	0.114
BMI Public university only, n = 519)^d^	24.3 (4.6)	24.3 (4.4)	24.6 (4.7)	24.4 (5.4)	0.976

^a^Calculated as the sum of intensity scores across pain sites (score of 0–20).

^b^The first line represents the mean (standard deviation) of the number of pain sites for each class.

^c^Rated on a score from none (0) to extreme (4). The first line represents the mean (standard deviation) of the pain intensity for each class at that region. The subsequent lines represent the distribution among the five pain intensity categories.

^d^Only age range was available from the medical university included in the study; therefore, age and BMI are only reported from the large public university participants.

### Mental Health Outcomes

#### Women

There was a significant difference between the “no pain” class and the other three classes for depression, anxiety, and stress (Table 4). In all cases, the scores from the DASS-21 were lowest in the “no pain” class (p < .021). The “moderate-severe multisite pain” class was also significantly different from the “mild multisite” class (p < .004), with scores being higher for the “moderate-severe multisite pain.” The differences between “moderate spine pain” and the other pain classes were inconsistent. Anxiety scores were higher in the “moderate spine pain” class than the “mild multisite pain” (p = .017) class while depression (p = .087) and anxiety (p = .083) did not differ. Depression scores were higher in the “moderate-severe multisite pain” class compared to the “moderate spine pain” class (p = .021), but anxiety (p = .073) and stress (p = .301) did not differ.

#### Men

There was a significant difference between the “no pain” class and the “mild” (p < .005) and “moderate multisite pain” (p < .001) classes, with depression, anxiety, and stress scores being lower in the “no pain” (Table 4). The “moderate multisite pain” class was significantly different from the “mild multisite” (p < .008) and the “mild extremity pain” (p < .002) classes, with scores being higher in the “moderate multisite pain”. Finally, the only difference that existed between the “mild multisite pain” class and the “mild extremity pain” class was for anxiety (p = .030), with anxiety scores being lower in the “mild extremity pain” class. There were no differences between the “no pain” class and the “mild extremity pain” class for the mental health variables (p > .223).

**Table 4:** Mean (95% CI) comparison in mental health variables between classes weighted for probability

	Mild multisite	No pain	Moderate spine pain	Moderate-severe multisite
Women (n = 685)^+^				
Depression^*^	8.8^a^ (7.8, 9.8)	7.1^b^ (6.0, 8.1)	11.0 ^a^ (8.4, 13.6)	16.5^c^ (12.3, 20.7)
Anxiety^*^	7.6 ^a^ (6.8, 8.5)	5.9^b^ (5.1, 6.8)	10.5^c^ (8.0, 12.9)	14.3^c^ (10.7, 18.0)
Stress^*^	13.0^a^ (11.6, 14.5)	10.6^b^ (9.1, 12.1)	16.3^a,c^ (12.6, 20.1)	19.6^c^ (14.6, 24.5)
	**Mild multisite**	**No pain**	**Mild extremity**	**Moderate-severe multisite**
Men (n = 578)^+^				
Depression^*^	9.9^a^ (8.5, 11.2)	7.5^b^ (6.5, 8.5)	9.0^a,b^ (6.7, 11.2)	16.8^c^ (11.5, 22.1)
Anxiety^*^	7.1^a^ (6.1, 8.0)	4.8^b^ (4.1, 5.5)	5.1^b^ (3.8, 6.4)	12.6^c^ (8.8, 16.5)
Stress^*^	11.0^a^ (9.6, 12.5)	7.8^b^ (6.8, 8.9)	8.5^a,b^ (6.4, 10.6)	17.3^c^ (12.0, 22.6)

^+^Overall contrast between classes p-value < 0.001 for all models. Different superscript letters indicate statistical difference between classes (p < .05). Classes are organized left to right by the largest class to the smallest class.

^*^Scores range from 0 to 42, with higher numbers indicating higher symptoms.

### Physical activity

#### Women

There were no significant differences between the four classes for total PA (Table 5); however, classes differed when separate work and leisure domains of physical activity were examined. Work physical activity was lowest in the “no pain” class compared to the “mild multisite” (p = .024), “moderate spine” (p = < 0.001) and “moderate-severe pain” (p < .001) classes. The “mild multisite” class had the second lowest work physical activity, which was also less than the “moderate spine” (p = .015) and “moderate-severe multisite” (p = .005) classes. There were no differences in work physical activity between the “moderate spine” and “moderate-severe multisite” (p = .636) classes. Leisure PA was highest for the “no pain” class, but only statistically different from the “moderate spine pain” class (p < .001), which had the lowest leisure PA of all classes.

#### Men

There were no significant differences between the four classes for total PA (Table 5); however, classes differed when separate work and leisure domains of physical activity were examined. Work physical activity was highest in the “moderate multisite” class compared to the “mild multisite” (p < .001), “no pain” (p = .001), and “mild extremity” (p = .001) classes. Leisure PA was lowest for the “mild multisite pain” class, and this was significantly different from the “no pain” (p = .012) and “moderate multisite pain” (p = .004) classes.

### Sleep

#### Women

Sleep scores were lowest, indicating better quality sleep, in the “no pain” class compared to the “mild multisite” (p = .010), “moderate spine” (p = .006), and “moderate-severe multisite” (p < .001) classes (Table 5). Sleep scores were highest in the “moderate-severe multisite” class compared to the “mild multisite” (p < .001) and “moderate spine” (p < .001) classes. There were no differences between the “mild multisite” and “moderate spine” pain classes (p = .251).

#### Men

Sleep scores were lowest in the “no pain” class compared to the “mild multisite” (p < .001) class (Table 5). Sleep scores were highest in the “moderate multisite” class compared to the “mild multisite” (p < .001), “no pain” (p < .001), and “mild extremity” (p < .001) classes. There were no differences between the “mild extremity” and “mild multisite” (p = .071) or “no pain” (p = .392) classes.

**Table 5:** Mean (95%CI) comparison in physical activity and sleep between classes weighted for probability

	Mild multisite	No pain	Moderate spine	Moderate-severe multisite
Women (n = 684)^+^				
Work Physical Activity (METmin/wk)	910.7^a^ (814.4, 1007.0)	745.9^b^ (643.3, 848.5)	1241.0^c^ (961.2, 1520.8)	1346.2^c^ (1008.4, 1684.1)
Leisure Physical Activity (METmin/wk)	1298.6^a^ (1160.7, 1436.5)	1545.2^a^ (1331.7, 1758.7)	943.0^b^ (729.0, 1157.1)	1251.7^a,b^ (935.4, 1568.0)
Total physical activity (METmin/wk)	3436.4^a^ (3073.1, 3799.6)	3446.0^a^ (2972.4, 3919.5)	3298.6^a^ (2555.0, 4042.2)	4096.0^a^ (3068.3, 5123.7)
Sleep^*^	5.7^a^ (5.4, 6.1)	5.0^b^ (4.5, 5.4)	6.2^a^ (5.4, 6.9)	8.6^c^ (7.7, 9.5)
	**Mild multisite**	**No pain**	**Mild extremity**	**Moderate-severe multisite**
Men (n = 584)^+^				
Work Physical Activity (METmin/wk)	644.3^a^ (562.2, 726.4)	686.5^a^ (598.9, 774.1)	631.9^a^ (481.5, 782.3)	1193.8^b^ (836.1, 1551.6)
Leisure Physical Activity (METmin/wk)	1258.1^a^ (1097.5, 1418.7)	1587.1^b^ (1383.3, 1790.9)	1400.6^a,b^ (1065.5, 1735.6)	2030.5^b^ (1422.2, 2638.7)
Total physical activity (METmin/wk)	3563.2 ^a^ (3109.5, 4016.8)	3802.8 ^a^ (3317.7, 4287.8)	3706.0 ^a^ (2824.7, 4587.4)	4920.8^a^ (3446.9, 6394.8)
Sleep^*^	6.0 ^a^ (5.6, 6.4)	4.8 ^b^ (4.4, 5.2)	5.2^b^ (4.4, 6.0)	8.4^c^ (7.4, 9.5)

Note: Overall contrast between classes p-value < 0.001 for women work METmin and sleep, and p = .002 for Men work METmin; Woman total PA p = .591 and male total PA p = .281; Woman leisure PA p = .003 and men leisure PA p = .009

^+^Different superscript letters indicate a statistical difference between classes (p < .05). Classes are organized from left to right by the largest class to the smallest class.

*Sleep scores from Pittsburgh Sleep Quality Index with higher scores indicating poorer quality sleep.

## Discussion

This cross-sectional study investigated unique musculoskeletal pain patterns, mental health outcomes, physical activity, and sleep among emerging adults (18–29 years). The first objective was to identify patterns of self-reported pain across multiple sites. We identified four classes of pain patterns separately for women and men. Both men and women had “no pain,” “mild multisite,” and “moderate-severe multisite” classes; however, their fourth class differed. Women had a “moderate spinal pain” class, while men had a “mild extremity pain” class. The second objective was to describe the identified classes for mental health outcomes, physical activity, and sleep and determine if differences exist between the classes. For both men and women, the “moderate-severe multisite” pain classes reported the highest levels of depression, anxiety, and stress, higher work physical activity, and poorer sleep than the “no pain” class. The “mild multisite” and “moderate spine pain” (women only) classes tended to fall between the “no pain” and “moderate/severe multisite” pain classes. The “mild upper extremity” pain class characteristics for men were very similar to their “no pain” counterparts.

Pain reporting within the last three months was substantial – 72% of women and 60% of men were categorized into one of the three pain classes. The largest class for our studies was “mild multisite pain” (50% of women and 40% of males). Previous reports for American adults report 35–40% of emerging adults report pain for at least one site over the past thirty days [42], and a European sample reported about one-third of boys and one-half of girls reported pain in the last 6 months [43]. The difference in reports could be because of how we asked about pain. The National Health Information Survey asked if they had “any symptoms of pain, aching, or stiffness in or around a joint over the past 30 days” [42], which is a “yes” or “no” question that does not take into account intensity. The North Finland Birth Cohort study asked people to consider their pain during the last six months, again without consideration for intensity [43]. Providing the location and intensity answer all in one question may have prompted more individuals to report mild pain than would have reported it if they were asked a “yes” or “no” question. Pain intensity is important to assess because can identify individuals with mild pain who would benefit from interventions that would prevent higher-intensity pain in the future.

Single-site pain reporting was not common in our sample (25% and 30% of women and men with reported pain, respectively), which is in line with previous literature [12, 27, 29]. The “mild multisite pain” locations for women were predominantly the shoulder/neck and low back, while for men, it was shoulder/neck, back, and knee. The “moderate-severe multisite” pain locations were again highest for the shoulder/neck and back for both men and women. The presence of low back pain and neck pain existing together within classes is supported by previous work that found shoulder/neck pain to be comorbid with low back pain [8]. In emerging adult aged workers, a class of individuals with simultaneous neck/back pain was also found using latent class analysis [30]; however, their analysis did not include pain intensity; thus, it could not decipher between mild or moderate/severe pain in those regions.

The fourth class difference between men and women could explain why the literature identifies differences in pain reporting between women and men. The fourth class for women was “moderate spinal pain,” where pain intensity was much higher for the shoulder/neck and low back than the “mild multisite” class. This aligns with previous work that found neck pain [8, 44–47], low back pain [14], and co-morbid neck and low back pain [8, 14] is higher in women than men. Since low back pain can impact activities of daily living [14], higher intensity and/or spine-related pain may be more related to lifestyle behaviors and outcomes in emerging adults than extremity pain alone, which is why there are more differences between the “no pain” classes and classes that contain mild or moderate spinal pain than extremity pain alone (as seen for men). The separation of women into two spinal pain classes and the lack of a “moderate spinal pain” class for men may be because some women could have symptoms of dysmenorrhea, which can include cramping pain felt in the low back [48]. Unfortunately, we did not ask about menstrual-related pain, and future studies on musculoskeletal symptoms in emerging adults should consider asking about such symptoms. The lack of a “moderate spinal pain” class for men may also be due to gender norms. Men may be more likely to tolerate or deny pain, while women may be more “sensitive,” be more willing to report their pain, and societal norms demonstrate acceptance for women to show and talk about pain [49]. Despite these norms, the percentage of men and women samples who reported “moderate-severe multisite pain”, was 7% and 9%, respectively, suggesting that men are still willing to report high-intensity pain via survey responses. Future work on pain development and sex/gender should use a mixed methods approach (quantitative and qualitative) to assess the biopsychosocial aspects of pain to comprehensively assess the different factors that influence pain and pain reporting.

Mental health outcomes in our sample became progressively higher from “mild multisite” to “moderate-severe multisite” pain. This is in line with previous work on adolescents. Adolescents are at risk for co-morbid low back pain and psychological disorders [4], and long-term multisite musculoskeletal pain from ages 16 to 18 is associated with anxiety and psychological distress in both genders [43]. Physical and mental health-related quality of life scores are lower in individuals classified as having high low back pain and light impact on activities of daily living from adolescence through early emerging adulthood [14]. Mental health outcomes modulate the relationship between other lifestyle behaviors and musculoskeletal pain, such that a lifestyle behavior may only cause pain if mental health outcomes are also poor [26]. Taken together, this information suggests that having multisite pain and higher pain intensity may be related to mental health and should be addressed simultaneously if an individual initially visits health professionals for just one of these issues.

While total physical activity did not differ between the classes, domain-specific physical activity demonstrated differences. For men, the “moderate-severe multisite pain” class was the only class with higher work physical activity, while for women, work physical activity was highest for the “moderate spinal” and “moderate-severe multisite” pain classes. For workers with low back pain, increasing moderate to vigorous physical activity at work by decreasing other work behaviors lead to a higher risk for long-term sickness [50]. A portion of our sample was also from a medical university. High job demands are associated with high musculoskeletal pain in those with direct patient care responsibilities and support staff [51], and those with low supervisor support report increased pain, especially women and nurses [51].

Leisure physical activity differences between the classes were also found for men and women. For men, leisure physical activity did not differ between the “moderate-severe multisite” and “no pain” classes, whereas it was higher for women. Differences in leisure time physical activity by gender is in line with global reports that women are less active than men [52], and physical inactivity trends are improving for adolescent boys but not girls [53]. Recent systematic reviews have found that occupational physical activity does not have a beneficial association with cardiovascular disease mortality [54] due to differences such as rest, duration, and intensity between these two types of physical activity[25]. For the men in the “moderate-severe multisite pain” class, the additive nature of the work and leisure physical activity may also mute the protective effects of physical activity [55]. To counter this, it is proposed that holistic changes can be made to both the work itself, leisure, and transport activities [56] or a balance of physical behavior over 24-hours[57]. Future work must continue to look at the different domains of physical activity when characterizing the impact of physical activity on health.

Poor sleep was evident in the “mild,” “moderate spinal,” and “moderate-severe multisite” pain classes compared to the “no pain” class, with both men and women in the “moderate-severe multisite” pain class having the poorest scores. In emerging adults, poor sleep is associated with higher levels of musculoskeletal pain and predicts an increase in pain severity three years later [23]. Sleep problems and daytime tiredness are associated with persistent pain from adolescence into emerging adulthood, especially for girls [7]. There is also a modest risk to adolescents who seek out primary care initially for sleep problems that they will return for musculoskeletal complaints in the future [6]. In relation to other factors, comorbid musculoskeletal pain and insomnia are associated with higher symptoms of anxiety and depression than those with only musculoskeletal pain [28]; however, rather than the hypothesis that sleep directly causes musculoskeletal pain, the potential path may be that inadequate sleep leads to poorer mental health, which then causes multisite chronic pain [26].

A main limitation of the study is the use of convenience samples and the cross-sectional study design, which limits results to associations and not causations between musculoskeletal pain and mental health. Its cross-sectional nature also limits the ability to examine the potential mediation of health behaviors (physical activity and sleep) on the relationship between pain and mental health. The sample of men in our study was 90% from the large public university, with only 67 participants from the medical university. This may have affected the lifestyle factor differences in our study for men. There was no significant difference in the percentage of individuals from the public versus medical universities between the classes; however, the highest percentage of individuals from the medical university was in the “moderate-severe pain” class for men (Table 3). For women, the percentage of individuals from the medical university was highest for the “moderate-severe pain” and “moderate spinal pain” classes (Table 2). Future work should consider studying employed 18-29-year-olds to assess the relationship between different domains of physical activity and musculoskeletal pain. Finally, we recruited individuals for a study on health, so it is possible that included participants may be biased; however, potential participants were unaware that the study would include questions about pain specifically before completing the survey.

## Conclusion

The latent class analysis identified four classes of pain location and intensity reporting in emerging adults (aged 18–29). For women and men, three of these classes represent “no pain,” “mild multisite pain,” and “moderate-severe multisite pain.” One of the classes differed between genders – men had a “mild extremity pain” class while women had a “moderate spinal pain” class. Pain reporting was substantial in our study, with 72% of women and 60% of men as part of the three pain-related classes. Classes with mild to moderate pain also had poorer mental health, physical activity, and sleep outcomes than the “no pain” and “mild extremity pain” classes. With the high reporting of mild multisite pain for both women and men, future work should determine if the pain intensifies later in life and a more detailed look to see if interventions that target emerging adults reduce the lifetime burden of musculoskeletal pain.

## Electronic supplementary material

Below is the link to the electronic supplementary material.

Supplementary Material 1

Supplementary Material 2

## Data Availability

The datasets used and/or analysed during the current study are available from the corresponding author upon reasonable request.
