# Supplementary material for: Musculoskeletal pain latent classes and biopsychosocial characteristics among emerging adults
Source: BMC Musculoskelet Disord. 2023 Apr 28;24:334. doi: 10.1186/s12891-023-06412-y (PMC10142412; doi:10.1186/s12891-023-06412-y)
Supplement: Supplementary file 1 — Supplementary Material 1 [file 12891_2023_6412_MOESM1_ESM.docx]

Additional File 1 Figure. Flow chart from original study sample to current study sample for secondary analysis.
