# Supplementary material for: Musculoskeletal pain latent classes and biopsychosocial characteristics among emerging adults
Source: BMC Musculoskelet Disord. 2023 Apr 28;24:334. doi: 10.1186/s12891-023-06412-y (PMC10142412; doi:10.1186/s12891-023-06412-y)
Supplement: Supplementary file 2 — Supplementary Material 2 [file 12891_2023_6412_MOESM2_ESM.docx]

**Supplemental Table 1a: Latent class model statistics for women.**

|  | **1-cluster** | **2-cluster** | **3-cluster** | **4-cluster** | **5-cluster** | **6-cluster** |
| --- | --- | --- | --- | --- | --- | --- |
| Df | 694 | 688 | 682 | 676 | 670 | 664 |
| L2 (p) | 1217 (<.001) | 776.1 (.01) | 657 (.75) | 626 (.91) | 604 (.97) | 590 (.98) |
| X2 (p) | 219393 (<.001) | 10317 (<.001) | 3026 (<.001) | 2538 (<.001) | 2053 (<.001) | 2183 (<.001) |
| BIC | -3343 | -3744 | -3824 | -3815 | -3798 | -3772 |
| AIC | -170 | -599 | -706 | -725 | -735 | -737 |
| Entropy (r2) | 1 | .72 | .70 | .64 | .66 | .67 |
| Class errors | 0 | .09 | .11 | .17 | .16 | .17 |
| Smallest % in cluster |  | .40 | .16 | .10 | .01 | .01 |
| Avg posterior probability |  | .93, .88 | .9, .88, .87 | .83, .87, .70, .87 | .85, .87, .7, .85, .72 | .82, .87, .76, .85, .73, .77 |
| Odds of correct classification |  | 8.9, 11 | 6.8, 19.8, 35.1 | 5.4, 18.1, 13.1, 58.5 | 6.1, 18.1, 13.2, 51, 254.6 | 5.8, 18.1, 17.9, 51, 87.4, 331.4 |

P=.002 for bootstrapped 4 vs. 3 class models in women.

**Supplemental Table 1b: Latent class model statistics for men.**

|  | **1-cluster** | **2-cluster** | **3-cluster** | **4-cluster** | **5-cluster** | **6-cluster** |
| --- | --- | --- | --- | --- | --- | --- |
| Df | 584 | 578 | 572 | 566 | 560 | 554 |
| L2 (p) | 930 (<.001) | 610 (.17) | 522 (.93) | 493 (.99) | 476 (1) | 462.4 (1) |
| X2 (p) | 4737892 (<.001) | 250897 (<.001) | 4875 (<.001) | 3521 (<.001) | 3932 (<.001) | 3161 (<.001) |
| BIC | -2809 | -3090 | -3140 | -3130 | -3190 | -3085 |
| AIC | -237 | -545 | -621 | -638 | -643 | -645 |
| Entropy (r2) | 1 | .69 | .68 | .66 | .69 | .75 |
| Class errors | 0 | .08 | .11 | .14 | .14 | .12 |
| Smallest % in cluster |  | .47 | .10 | .09 | .08 | .05 |
| Avg posterior probability |  | .91, .93 | .92, .87, .84 | .88, .87, .75, .86 | .87, .87, .8, .87, .84 | .88, .88, .9, .9, .86, .77 |
| Odds of correct classification |  | 9.0, 15.0 | 10.3, 11.6, 44.7 | 10.8, 12.0, 17.0, 64.3 | 11.9, 15.9, 19.3, 71.9, 58.9 | 13.0, 26.1, 48.9, 55.6, 79.7, 59.2 |

P=<.001 for bootstrapped 4 vs. 3 class models in men.
